# Implementing reverse translational research in psychiatry

**DOI:** 10.3389/fpsyt.2026.1864491

**Published:** 2026-07-08

**Authors:** Annakarina Mundorf, Sebastian Ocklenburg

**Affiliations:** 1 ISM Institute for Systems Medicine and Department of Human Medicine, MSH Medical School Hamburg, Hamburg, Germany; 2 ICAN Institute for Cognitive and Affective Neuroscience and Department of Psychology, MSH Medical School Hamburg, Hamburg, Germany; 3 Biopsychology, Institute of Cognitive Neuroscience, Faculty of Psychology, Ruhr University Bochum, Bochum, Germany

**Keywords:** addiction disorders, animal models, anxiety disorders, behavior & cognition, mood disorder, reverse translational research

## Introduction

Animal models play an important role in psychiatric research, but a lack of translational relevance can impair their usefulness. For example, some rodent paradigms, including the forced swim and tail suspension test, have long been employed to study depressive- and anxiety-like behaviors. While widely used, their translational relevance remains limited. Forced swim and tail suspension paradigms, for instance, primarily measure immobility as a proxy for behavioral despair, but this behavior can be influenced by locomotor activity or habituation rather than core depressive features. Importantly, these paradigms are often interpreted within a broader behavioral test battery that includes measures such as locomotor activity or open field exploration to control for such confounds, although this does not fully resolve concerns about their construct validity. Recent work demonstrates that the forced swim test predicts antidepressant efficacy poorly, with only a fraction of compounds showing correspondence with human clinical outcomes (1). Historically, these assays were developed and validated primarily as pharmacological screening tools for monoaminergic antidepressants, which may further limit their utility for identifying novel mechanisms or treatments acting via non-traditional targets (1). These limitations create discrepancies between preclinical and clinical outcomes, reducing the predictive power of animal studies and underscoring the need for approaches that better link models with patient-relevant phenomena (2–9).

## Bridging the translational gap in psychiatry

The translational gap is amplified by psychiatric heterogeneity: patients with the same DSM diagnosis may differ in symptoms, trajectory, and treatment response, yet preclinical models often reduce disorders to uniform behavioral readouts.

**Figure 1:**
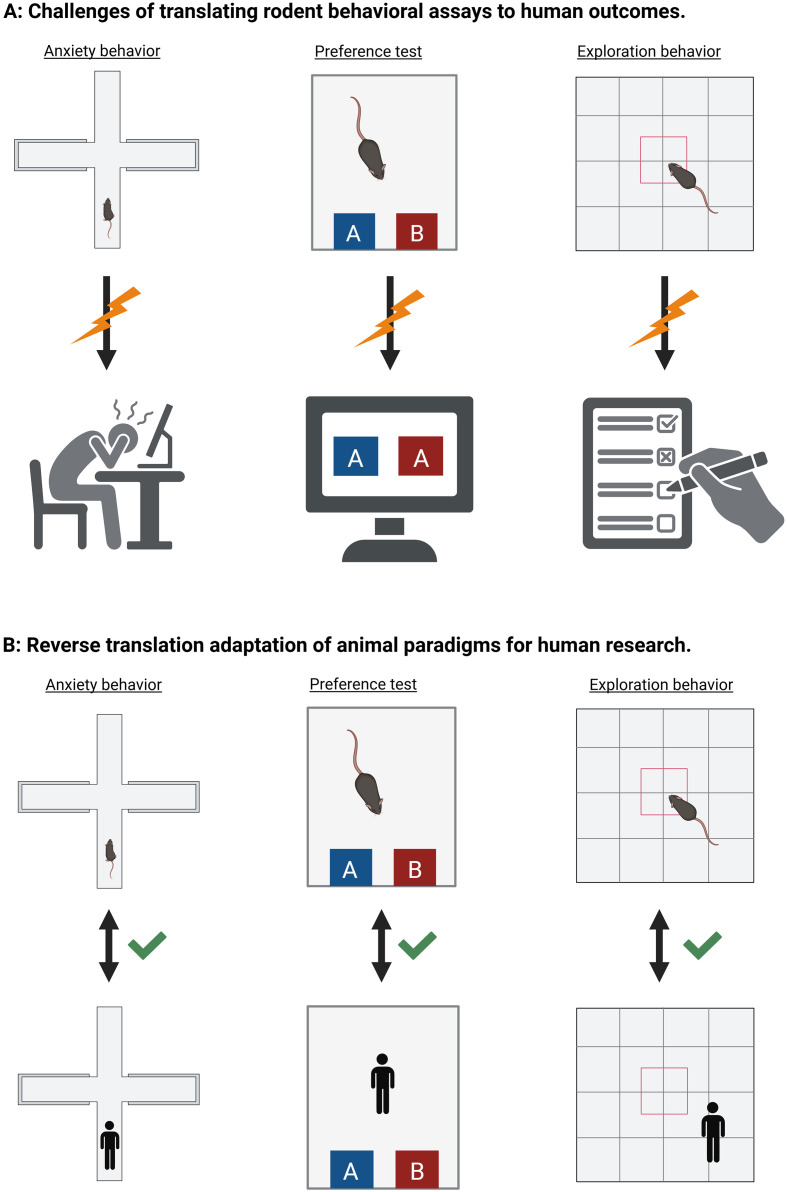
Conventional and reverse translational approaches for assessing behavioral constructs across species. **(A)** Rodent behavioral paradigms commonly used to assess anxiety, preference, and exploration (e.g., elevated plus maze, preference tests, and open field tests) are translated to human assessments that often differ substantially in task design and outcome measures. This mismatch can reduce cross-species comparability and limit the predictive value of preclinical findings. **(B)** In reverse translational research, paradigms established in animal research are translated to human applications to enhance cross-species comparability. This approach aims to maintain core experimental features across species, enabling more direct comparison of behavioral outcomes. For example, anxiety-related behavior could be assessed using a mixed-reality elevated plus maze adapted from the rodent paradigm. Created in BioRender. Mundorf, A (2026). https://BioRender.com/34qd8s4.

One new approach to achieve this is so-called reverse translational research. While the term “reverse translation” has been used inconsistently in the literature and alternative definitions have been proposed [e.g., (10–12)], it has also been used to describe approaches in which paradigms originally developed in animal research are adapted for use in humans to improve cross-species comparability [e.g., (2, 9)]. In translational research, an animal model is created to mimic symptom patterns experienced by human patients with a specific diagnosis. This way basic research on cellular or molecular processes in this disorder can be conducted in the animal model and results from these studies can provide insights into optimizing treatment in human patients. One problem with this approach is that the tasks used to assess symptoms of psychiatric disorders in these animal models are quite different than psychiatric diagnostics with clinical interviews in patients. Reverse translational research addresses this need by inverting the conventional bench-to-bedside approach: animal models and preclinical paradigms inform the design of human experiments, enabling more precise and comparable measures in clinical studies. In the present article, we use the term “reverse translation” in this latter sense, which serves as our operational definition, namely the adaptation of paradigms originally developed in animal research for application in humans, thereby facilitating more direct comparisons across species. By translating insights from established animal paradigms to humans, reverse translation enhances construct validity and improves interpretability across species. However, this does not imply that validity is automatically transferred from animal to human paradigms; instead, it must be evaluated separately in each species-specific implementation. Importantly, while cross-species approaches are not entirely new [classical conditioning and extinction paradigms have long been successfully implemented in both animals and humans (3, 13)], these approaches were often highly controlled and theory-driven, rather than grounded in ethologically relevant behavior. Reverse translational strategies extend this tradition by focusing more explicitly on naturalistic, species-relevant behaviors and their underlying neurobiological mechanisms. The rationale underlying reverse translational research is illustrated in **Figure 1**, which highlights how adapting animal paradigms for human use may facilitate more direct comparisons of behavioral outcomes across species.

## From animal paradigms to human experiments

Several recent studies illustrate reverse translational research by adapting well-established animal behavioral paradigms for humans. For example, a virtual reality (VR) version of the open field test showed that humans, like rodents, prefer to stay close to walls rather than explore the center. This behavior is enhanced in individuals with agoraphobia or high anxiety sensitivity, demonstrating the relevance of animal-derived measures for human anxiety (14). Similarly, a mixed-reality version of the rodent elevated plus maze revealed comparable avoidance of open arms and modulation of behavior by anxiety levels and pharmacological interventions (15). More broadly, ethologically informed animal paradigms can provide the conceptual and methodological foundation for designing human experiments and generating cross-species benchmark data (2, 16). Comparable cross-species alignment has also been demonstrated in probabilistic learning, affective bias, and effort-based decision-making paradigms (2), further supporting the generalizability of this approach across psychiatric domains. By emphasizing construct validity and translational relevance, these approaches allow preclinical behavioral tests to meaningfully inform human research, refining our understanding of anxiety and depressive phenotypes and guiding the development of clinically relevant assessments. In this context, construct validity should not be understood as mere surface similarity between animal and human behavior, but rather as the extent to which paradigms capture shared underlying processes or latent constructs that are measurable across species.

Beyond depression and anxiety, reverse translational strategies have been applied in other neuropsychiatric contexts. For instance, alcohol use disorder research demonstrates how well-characterized rodent paradigms can be translated into human laboratory experiments, clarifying which behavioral readouts correspond to clinical constructs such as compulsive consumption or reward sensitivity (3). By systematically aligning preclinical and clinical endpoints, these approaches facilitate the design of human studies directly informed by animal behavior and enhance predictive validity. However, in psychiatric research, these strategies remain largely underutilized, often restricted to isolated studies or theoretical comparisons of which animal tasks correspond to specific human endpoints.

## Discussion

Reverse translation has been used in translational research to adapt paradigms originally developed in animal models for use in humans and to link specific behavioral and cognitive functions across species (14, 15, 17). This approach is still not widely implemented in a systematic way, although it offers a way to study functional processes rather than complete psychiatric disorders. This is particularly relevant because animal models typically capture symptom-like behavior instead of full clinical syndromes, which limits disorder-level translation but allows functional comparisons across species. However, this functional perspective aligns with the Research Domain Criteria (RDoC) framework, which focuses on dimensional constructs rather than diagnostic categories (18, 19) and provides a useful structure for organizing cross-species research at the level of specific behavioral and neural systems. A key challenge for reverse translation is that many traditional animal paradigms cannot always be directly implemented in humans under comparable experimental conditions. Recent developments, especially VR, now make it possible to reproduce established animal paradigms in humans with higher experimental control and improved ecological validity.

To make reverse translation more systematic, we propose a stepwise implementation strategy:

1. Define the functional domain of interest, ideally based on RDoC constructs to ensure cross-species comparability at the level of specific behavioral or cognitive functions.
2. Select a validated animal paradigm that reliably captures the target function and has established behavioral and neurobiological validity.
3. Adapt the paradigm for human use, for example using VR, behavioral tasks, or neurophysiological measures such as EEG or fMRI, while maintaining construct overlap across species as closely as possible.
4. Empirically evaluate cross-species validity, including construct validity (functional equivalence), and when applicable, predictive or convergent validity across measures.
5. Iteratively refine the paradigm based on discrepancies between species-specific implementations, allowing bidirectional adjustment of task parameters. For example, if a rodent task relies on spatial exploration in an open field, VR parameters in humans (e.g., movement constraints or reward structure) may be adjusted when behavioral patterns do not show comparable exploratory dynamics.
6. Assess practical adequacy of the adaptation based on predefined criteria such as behavioral robustness, interpretability, and reproducibility across cohorts. For example, a VR-based adaptation of a fear conditioning task would be considered adequate if it reliably elicits avoidance behavior across participants, produces consistent physiological responses (e.g., skin conductance or heart rate), and shows stable effects across independent samples.

This structured approach shifts reverse translation from a largely conceptual idea toward an operational framework for study design. It may improve reproducibility, comparability, and translational value across studies. In summary, reverse translation combined with modern experimental tools such as VR provides a promising way to bridge animal and human research at the level of specific cognitive and behavioral functions.

## References

[1] Trunnell ER Carvalho C . The forced swim test has poor accuracy for identifying novel antidepressants. Drug Discov Today. (2021) 26:2898–904. doi: 10.1016/j.drudis.2021.08.003 34390862

[2] Gencturk S Unal G . Rodent tests of depression and anxiety: Construct validity and translational relevance. Cognit Affect Behav Neurosci. (2024) 24:191–224. doi: 10.3758/s13415-024-01171-2 38413466 PMC11039509

[3] Nieto SJ Grodin EN Aguirre CG Izquierdo A Ray LA . Translational opportunities in animal and human models to study alcohol use disorder. Transl Psychiatry. (2021) 11:496. doi: 10.1038/s41398-021-01615-0 34588417 PMC8481537

[4] Nestler EJ Hyman SE . Animal models of neuropsychiatric disorders. Nat Neurosci. (2010) 13:1161–9. doi: 10.1038/nn.2647 20877280 PMC3750731

[5] Silverman JL Nithianantharajah J Der-Avakian A Young JW Sukoff Rizzo SJ . Lost in translation: At the crossroads of face validity and translational utility of behavioral assays in animal models for the development of therapeutics. Neurosci Biobehav Rev. (2020) 116:452–3. doi: 10.1016/j.neubiorev.2020.07.008 32681939 PMC7773218

[6] Gyles TM Nestler EJ Parise EM . Advancing preclinical chronic stress models to promote therapeutic discovery for human stress disorders. Neuropsychopharmacol. (2024) 49:215–26. doi: 10.1038/s41386-023-01625-0 37349475 PMC10700361

[7] Robinson ES . Delivering a new generation of translational animal models for depression research. Behav Pharmacol. (2025) 36:175–81. doi: 10.1097/FBP.0000000000000819 40336488

[8] Tan VT Ward RD . Lost in translation: toward clinically effective translational research. Transl Psychiatry. (2025) 15:478. doi: 10.1038/s41398-025-03688-7 41249805 PMC12623474

[9] Mundorf A Ocklenburg S . Laterality of rodent behaviour: Why it matters for basic and clinical neuroscience and an outline for reverse-translational laterality research. Laterality. (2026), 1–28. doi: 10.1080/1357650X.2026.2679273 42204949

[10] Kangas BD . Reverse translation. Perspect Behav Sci. (2026) 49:9–26. doi: 10.1007/s40614-025-00478-w 41890686 PMC13013796

[11] Daniels S Horman T Lapointe T Melanson B Storace A Kennedy SH . Reverse translation of major depressive disorder symptoms: A framework for the behavioural phenotyping of putative biomarkers. J Affect Disord. (2020) 263:353–66. doi: 10.1016/j.jad.2019.11.108 31969265

[12] Peleh T Ike KG Wams EJ Lebois EP Hengerer B . The reverse translation of a quantitative neuropsychiatric framework into preclinical studies: Focus on social interaction and behavior. Neurosci Biobehav Rev. (2019) 97:96–1118. doi: 10.1016/j.neubiorev.2018.07.018 30660427

[13] Anselme P Güntürkün O . Connecting extinction learning in the laboratory and the wild. Nat Rev Psychol. (2026) 5:352–65. doi: 10.1038/s44159-026-00561-2 37880705

[14] Walz N Mühlberger A Pauli P . A human open field test reveals thigmotaxis related to agoraphobic fear. Biol Psychiatry. (2016) 80:390–7. doi: 10.1016/j.biopsych.2015.12.016 26876946

[15] Biedermann SV Biedermann DG Wenzlaff F Kurjak T Nouri S Auer MK . An elevated plus-maze in mixed reality for studying human anxiety-related behavior. BMC Biol. (2017) 15:125. doi: 10.1186/s12915-017-0463-6 29268740 PMC5740602

[16] Drzewiecki CM Fox AS . Understanding the heterogeneity of anxiety using a translational neuroscience approach. Cognit Affect Behav Neurosci. (2024) 24:228–45. doi: 10.3758/s13415-024-01162-3 38356013 PMC11039504

[17] Young JW Geyer MA Rissling AJ Sharp RF Eyler LT Asgaard GL . Reverse translation of the rodent 5C-CPT reveals that the impaired attention of people with schizophrenia is similar to scopolamine-induced deficits in mice. Transl Psychiatry. (2013) 3:e324. doi: 10.1038/tp.2013.82 24217494 PMC3849961

[18] Cuthbert BN Insel TR . Toward the future of psychiatric diagnosis: the seven pillars of RDoC. BMC Med. (2013) 11:126. doi: 10.1186/1741-7015-11-126 23672542 PMC3653747

[19] Insel T Cuthbert B Garvey M Heinssen R Pine DS Quinn K . Research domain criteria (RDoC): toward a new classification framework for research on mental disorders. Am J Psychiatry. (2010) 167:748–51. doi: 10.1176/appi.ajp.2010.09091379 20595427

